# Motion Sensors-Based Machine Learning Approach for the Identification of Anterior Cruciate Ligament Gait Patterns in On-the-Field Activities in Rugby Players

**DOI:** 10.3390/s20113029

**Published:** 2020-05-27

**Authors:** Salvatore Tedesco, Colum Crowe, Andrew Ryan, Marco Sica, Sebastian Scheurer, Amanda M. Clifford, Kenneth N. Brown, Brendan O’Flynn

**Affiliations:** 1 Tyndall National Institute, University College Cork, Lee Maltings Complex, Dyke Parade, T12R5CP Cork, Ireland; colum.crowe@tyndall.ie (C.C.); marco.sica@tyndall.ie (M.S.); brendan.oflynn@tyndall.ie (B.O.); 2 School of Allied Health, Health Research Institute, University of Limerick, V94T9PX Limerick, Ireland; ryana27@tcd.ie (A.R.); Amanda.Clifford@ul.ie (A.M.C.); 3 Insight Centre for Data Analytics, School of Computer Science and Information Technology, University College Cork, T12XF62 Cork, Ireland; sebastian.scheurer@insight-centre.org (S.S.); k.brown@cs.ucc.ie (K.N.B.)

**Keywords:** machine learning, ACL, biomechanics, IMUs, inertial sensors, gait analysis, running, on-the-field, rugby

## Abstract

Anterior cruciate ligament (ACL) injuries are common among athletes. Despite a successful return to sport (RTS) for most of the injured athletes, a significant proportion do not return to competitive levels, and thus RTS post ACL reconstruction still represents a challenge for clinicians. Wearable sensors, owing to their small size and low cost, can represent an opportunity for the management of athletes on-the-field after RTS by providing guidance to associated clinicians. In particular, this study aims to investigate the ability of a set of inertial sensors worn on the lower-limbs by rugby players involved in a change-of-direction (COD) activity to differentiate between healthy and post-ACL groups via the use of machine learning. Twelve male participants (six healthy and six post-ACL athletes who were deemed to have successfully returned to competitive rugby and tested in the 5–10 year period following the injury) were recruited for the study. Time- and frequency-domain features were extracted from the raw inertial data collected. Several machine learning models were tested, such as k-nearest neighbors, naïve Bayes, support vector machine, gradient boosting tree, multi-layer perceptron, and stacking. Feature selection was implemented in the learning model, and leave-one-subject-out cross-validation (LOSO-CV) was adopted to estimate training and test errors. Results obtained show that it is possible to correctly discriminate between healthy and post-ACL injury subjects with an accuracy of 73.07% (multi-layer perceptron) and sensitivity of 81.8% (gradient boosting). The results of this study demonstrate the feasibility of using body-worn motion sensors and machine learning approaches for the identification of post-ACL gait patterns in athletes performing sport tasks on-the-field even a number of years after the injury occurred.

## 1. Introduction

Over 200,000 anterior cruciate ligament (ACL) injuries occur in the USA alone annually, with more than half of these injuries requiring surgical reconstruction and subsequent rehabilitation [1]. This number is expected to increase in the coming years [2]. The highest growth of ACL injuries is in young and active people (under 25 years) [2] which, in the long term, may increase the risk of developing osteoarthritis and disability (e.g., reduced performance of daily living activities, leisure time activities, or sports activities). In particular, the rise of ACL injury in young people has been attributed to earlier specialisation by younger athletes, longer sporting seasons, more intense training, higher levels of competition, and a lack of free play [2].

After surgical reconstruction, patients aiming to return to sport (RTS) go through a pre-defined rehabilitation programme which is deemed successful if the patient is able to return to the same level of sporting activity as before the injury [3]. However, on average, 80% of patients were found to return to sport, with only 55% returning to competitive levels after ACL reconstruction [4]. In professional sport, the RTS rate in professional male soccer players was higher (90%) one year after ACLR, but only 65% were still playing at the highest level three years after ACL reconstruction [5]. 

King et al. [6] highlighted that biomechanical differences between ACL and non-ACL reconstructed knees were evident at nine months after surgery despite no difference in performance time during a change-of-direction (COD) task. As shown by Slater et al. [7], alterations in frontal- and sagittal-plane walking kinematics and kinetics observed early (<12 months) after surgery persisted in the following period (12–36 months). Despite clearance to return to physical activity, these gait patterns do not appear to normalize over time, which may indicate that the current approach to rehabilitation and assessment before return to activity is not adequate in identifying individuals with dysfunctional movement patterns. This was also confirmed in [8,9,10,11], where differences in joint kinematics and gait pattern (i.e., in anteroposterior translation and laxity, hamstring muscle activation, eccentric knee flexor strength) were also observed up to 5–10 years following reconstruction in subjects whose rehabilitation was deemed successful and returned to sport. 

These results indicate that RTS still represents an important challenge after ACL reconstruction and that current clinical and sport practices could be improved. Indeed, no consensus exists on the most appropriate criteria for RTS after ACL, and criteria typically considered by clinicians involve time after surgery, self-reported patient-outcome measures (such as the International Knee Documentation Committee - IKDC questionnaire), clinical examination (e.g., muscle strength, hop tests, limb symmetry, movement quality, fatigue), and psychological factors [5]. Indeed, Dingenen and Gokeler [5], state current RTS measures are predominantly subjective and recommend we use more evidence-based objective RTS criteria. At present, there is a dearth of evidence supporting the relationship between RTS and standard subjective and objective assessments, which questions if existing RTS assessments and criteria are sensitive or demanding enough to elucidate clinically relevant indicators.

While marker-based motion analysis systems (e.g., Vicon) [12] can provide objective assessments and represent the gold-standard technology adopted in gait analysis for quantitative movement analysis, their adoption is constrained by cost, access to specialist motion labs, as well as the practicality of application for larger patient/subject groups and, thus, shows limited use for on-the-field players. The market for wearable sensors has been growing significantly in recent years and such technologies represent a viable alternative to gold-standard technologies, offering remote real-time objective assessment at low cost and with small size. 

The adoption of wearables in sport has spread significantly in individual athletes, sports teams, and physicians for the possibility to monitor in real-time functional movements, workloads, and biometric markers during training and competitive sports [13]. Moreover, the integration of machine learning and artificial intelligence with wearable technology, thanks to the vast amount of data available nowadays, yields promising results in terms of athletic performance, coaching support, technique correction, injury prediction, and so on. Some relevant examples of the use of machine learning with inertial sensors in sport include [14,15,16,17]. 

A number of studies have considered the application of inertial sensors during ACL rehabilitation in laboratory settings [18,19,20], however few works have evaluated the performance of athletes who returned to sport using wearable technology. 

For example, Patterson et al. [21] investigated 14 athletes post-ACL reconstruction (an average of 3.5 years after surgery) and 17 athletes as a control group in a walking scenario using two inertial sensors on the shanks and highlighted that gyroscope features were able to discriminate healthy from ACL-reconstructed individuals, which was not possible using gait temporal variables. On the contrary, Setuain et al. [22,23] tested 26 elite handball players (six of them were ACL-reconstructed with an average time since surgery of 6.3 years) using an inertial sensor worn on the lower-back during horizontal and vertical jumping tasks. However, no difference was evident among the two groups. Finally, Mandalapu et al. [24] applied motion sensors and machine learning models on 131 subjects (109 of them with ACL injury) to discriminate between the two classes with good results. However, the tests were carried out in a lab setting and the time since surgery was not provided.

It is evident that additional testing of wearable sensor technologies used by athletes on-the-field after RTS is needed. The aim of this study is two-fold:

(i) to investigate whether there is a long after-effect of the ACL damage in rugby players, detecting significant differences in ACL-reconstructed vs. healthy players, when involved in a change-of-direction activity;
(ii) to provide an automated and objective method to distinguish between healthy and post-ACL groups of rugby players which is independent from subject-related information, step detection and segmentation processes, and standard gait spatiotemporal metrics, through the combination of a set of inertial sensors worn on the lower-limbs and data-driven machine learning models.

To the best of the authors’ knowledge, the combination of a data-driven approach and inertial sensors to classify healthy and ACL-reconstructed subjects on-the-field (with post-ACL athletes returned to sport and with time from surgery between five and 10 years) is not yet explored. 

## 2. Materials and Methods

### 2.1. Participants

The analysis in this study is based on a sample of twelve non-elite rugby players (all males, age: 26 ± 5.2 years; height: 182.6 ± 5.8 cm; mass: 90 ± 12.8 kg). Players were recruited via a general invitation e-mail, posters, and word of mouth, to students at a University in Ireland. 

The subjects were divided in two groups: six players with a history of ACL reconstruction surgery (age: 29.3 ± 4.5 years; height: 182.3 ± 6.2 cm; mass: 89.2 ± 14.7 kg), and six players with no history of lower-limbs injuries (age: 22.8 ± 3.7 years; height: 182.8 ± 6.1 cm; mass: 90.8 ± 11.9 kg). All the subjects who underwent ACL surgery were deemed successfully recovered by their consultant and had returned to play rugby competitively. Surgery occurred between 5 and 10 years before data collection. All injuries occurred on the left leg of the subjects with a history of ACL surgery and one player who was re-injured underwent a second repair on the left leg.

Prior to participation, volunteers received a verbal and written explanation of the study protocol and written consent was obtained. Socio-demographic information was collected on gender, age, weight, height, previously injured leg, time since surgery, and position on the field. Summary information on the subjects are displayed in Table 1. The study received ethical approval from the University’s Research Ethics Committee. 

### 2.2. Data Collection Protocol

**Figure 1:**
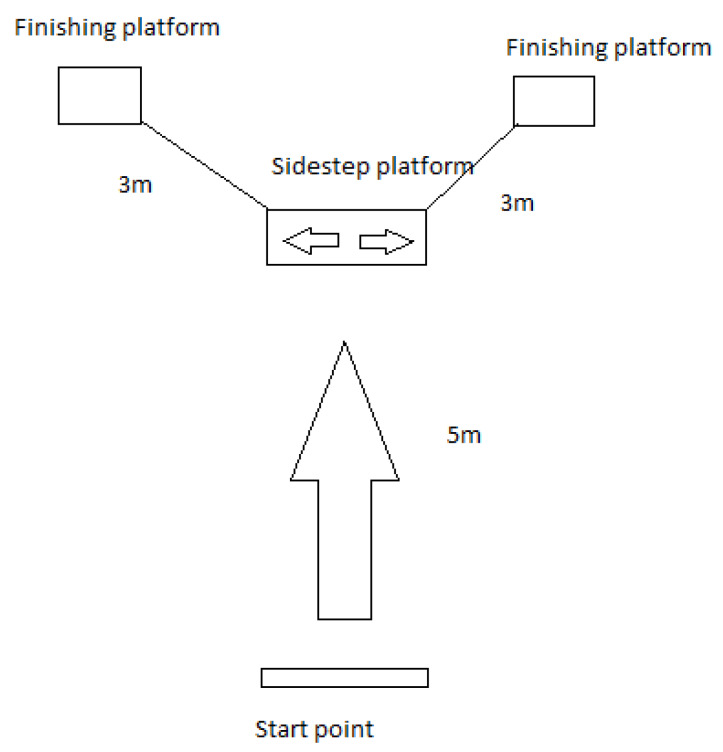
Experimental design.

Both cohorts performed the same task, summarized in Figure 1. Each participant began the data collection at a pre-defined start point, and was asked to run for 5 m towards a side-step platform. During the run, the participants were instructed regarding which direction the side-step had to occur (left or right). They were then required to step at a 45 degree angle from the sidestep board in either direction, and finally run an additional 3 m to come to a full stop. Sidestepping was considered as it represents a demanding and characteristic movement in rugby, which is associated with a higher risk of ACL injury [25]. The test was performed at maximum speed to replicate as closely as possible the knee cutting maneuvers performed in real-world matches. Each subject repeated the test ten times, with five sidestepping to the left and five to the right. The sequence of the sidestepping direction was randomized using a computer-generated sequence. The volunteers could rest as long as they required between the repetitions to avoid fatigue. 

**Figure 2:**
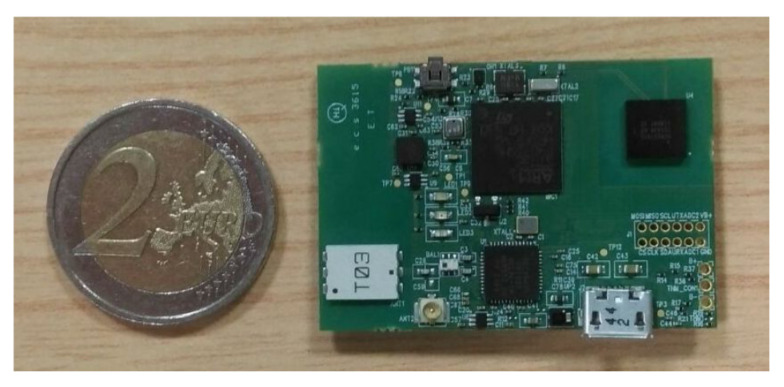
Hardware platform used for data collection.

The hardware platforms described in [19] were adopted for data collection (Figure 2). Two inertial measurement units (IMUs) were attached per leg, in particular to the anterior tibia, 10 cm below the tibial tuberosity, and to the lateral thigh, 15 cm above the tibial tuberosity, using Velcro straps. These IMUs were custom-made and equipped with a high-performance, low-power 168 MHz 32-bit microprocessor with 1 MB of flash memory and 192 KB + 4 KB of random access memory (RAM), a Bluetooth low-energy (BLE) communication module, a rechargeable battery, sensors for barometric pressure, humidity, temperature, and a tri-axial accelerometer, gyroscope, and magnetometer. For this study, only data from the accelerometer and the gyroscope were collected using a MPU-9250 with sensing range of 16 g and 2000 deg/s, respectively. The inertial sensors connect to the micro-controller over the Inter-Integrated Circuit (I2C) bus. Each platform measures 44 × 30 × 8 mm, weighs 7.2 g without battery (and 40 g including battery and enclosure), and collects synchronized three-dimensional accelerometer and gyroscope data sets at 100 Hz which are stored on a micro SD card. Before each repetition, subjects were asked to perform a deep squat in order to temporally synchronize the beginning of the test among all the sensors worn by the participant. 

Data collection occurred at University’s playing pitches between September and December 2016.

### 2.3. Preliminary Data Processing

**Figure 3:**
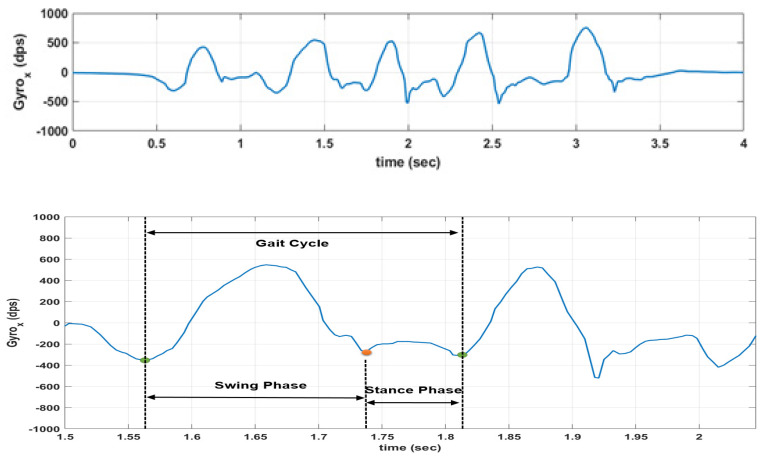
Example of raw inertial data (angular rate over the sagittal axis) collected for one repetition from a sensor on the shank, and the definition of gait cycle, stance and swing phases. Stance phase, swing phase, and gait cycle are marked with dotted lines.

The raw inertial signals collected by the four sensors in each repetition were temporally aligned using the characteristic movements associated with the deep squat movement carried out for this purpose at the beginning of the test and then re-sampled to a common sampling rate in order to take into account possible fluctuations in the sampling rates. Also, in order to have the same reference system for both IMUs worn on the same leg, the method proposed by Seel et al. [26] has been adopted to virtually rotate along the horizontal axis the raw inertial data recorded by the sensors worn on the shank. As a result, for all the IMUs involved, the *x*-axis represents the mediolateral axis, the *y*-axis is the anteroposterior one, while the *z*-axis is the vertical axis. Thus, the plane y–z represents the sagittal plane. Figure 3 shows an example of the inertial data collected from one of the sensors during one repetition.

Owing to technical issues with system hardware during data recording, six data collections out of the overall 120 (e.g., 5% of the original dataset) were not available for data processing. Of these six, four occurred in the cohort of subjects post-ACL and two in the healthy control group. As such, 58 data sets from uninjured players and 56 data sets from post ACL players were available for the study. 

Using the inertial data collected from the sensor on the calf and the method described in [27], a number of temporal gait parameters were extracted for each step for both legs during every repetition. Those gait parameters include gait cycle time (GCT), stance phase (STP), swing phase (SWP), relative STP and SWP (rSTP and rSWP, defined as the ratio between STP and GCT, and SWP and GCT, respectively), and cadence (defined as the inverse of the GCT). A two-way ANOVA was implemented for each gait parameter for a comparison between the healthy and the post-ACL groups taking into account all the steps extracted during every repetition. Significance value was set at *p* = 0.05 for all statistical analysis. 

Preliminary data processing was carried out in MATLAB R2017b (Mathworks, Inc., Natick, MA, USA) while statistical analysis was performed in IBM SPSS^®^ statistics software 25.0 Version (IBM SPSS^®^ Statistics, Armonk, New York, NY, USA).

### 2.4. Feature Extraction 

From the data collected for each repetition, a number of features were extrapolated. The signals considered for feature extraction were the 3D angular rate, the magnitude of the 3D acceleration, the 3D jerk signal obtained from differentiation of the 3D acceleration, and the 3D acceleration in the body-frame and gravity-frame. The 3D acceleration in the body and gravity frames were obtained from the measured 3D acceleration via the method discussed in [28]. The following statistical features were obtained by all these signals: mean, standard deviation, skewness, kurtosis, root mean square (RMS), minimum, maximum, peak-to-peak, coefficient of variation (CV), and interquartile range (IQR). Pairwise correlation, signal energy, and signal magnitude area (SMA) were also extracted, but not from the magnitude of the acceleration.

Frequency-domain features, such as dominant frequency, spectral centroid, spectral edge frequency, harmonic ratio, and index of harmonicity were obtained from the 3D angular rate, 3D acceleration (without distinction between body- and gravity-frame), 3D jerk, and acceleration magnitude. 

Moreover, a dimensionless jerk-based smoothness measure was also obtained [29]. Finally, sample entropy [30] and smoothness measure-related spectral arc length (SPARC) [31] were also obtained from the 3D gyroscope and accelerometer.

More information on those features can be found in [32,33]. Overall, 250 features were therefore obtained from each repetition (Supplementary Table S1). This analysis did not include subject-related features (e.g., height, weight) in order to develop subject-independent models based exclusively on data collected from the wearable sensors and the movement mechanics. Feature extraction was performed in MATLAB R2017b (Mathworks, Inc., Natick, MA, USA).

### 2.5. Learning Model

Several supervised-based classifiers were developed in Python 3 (Python Software Foundation, Delaware, US) for analysis. Models considered for the classifier were k-nearest neighbors (kNN), naïve Bayes (NB), support vector machine (SVM), gradient boosting tree (XGB), multi-layer perceptron (MLP), and stacking [34]. Accuracy was used as the metric to quantify the goodness-of-fit comparing the predictions of the classifier with the real labels each observation belonged to. Accuracy was computed for each player and then the mean and standard error (SE) of the accuracy were obtained from all the players in the training, validation and test datasets.

For model building, the dataset was randomly split into training (6 subjects), validation (2 subjects) and test sets (4 subjects), accounting for roughly 50%, 17%, and 33% of the total sample size, respectively. Stratification was taken into account during the partitioning, thus guaranteeing that training, validation, and test datasets are obtained with the same number of players belonging to both cohorts (healthy group, and post-ACL). 

A grid search was employed on the training set to attain optimal values for the model hyper-parameters (the full list of hyper-parameters evaluated in the grid search is available in the Supplementary Table S2). Model fitting and feature selection (select K best with K ≤ 10 to avoid overfitting) were deployed simultaneously. For each combination of hyper-parameters’ values, a leave-one-subject-out cross-validation (LOSO-CV) was carried out. Mean accuracy and SE were obtained from the 6 subjects in the training dataset. The combination of hyper-parameters that returned the best accuracy was considered as the optimum and the selected model was evaluated on the validation set to prove its generalizability. Consecutively, training and validation sets were merged into a single new training set (8 subjects) and the model was re-trained via LOSO-CV to determine the final optimal hyper-parameters and features, and accuracy was obtained for the 4 subjects in the test dataset, from which average accuracy and SE were calculated. Regularization was also included to avoid any potential overfitting.

LOSO-CV was adopted as it is well-reported in the literature that this approach is the most appropriate cross-validation method to estimate the generalizability of a model for an unseen user who is not included in the training data, especially if the dataset is characterized by a limited number of subjects, providing a more conservative and unbiased indication on the model performance [35].

To guarantee that players’ partitioning in the training, validation, and test datasets does not impact the model’s results, the method is repeated 10 times with random assignments of the players to the three training-validation-testing datasets (with the stratification constraint always maintained) and the mean accuracy and SE over the 10 permutations is finally reported. 

## 3. Results

### 3.1. Gait Analysis Results

A two-way ANOVA was performed to observe potential significant differences in temporal gait parameters between the post-ACL group and the control group. A summary of the descriptive statistics for each subgroup is shown in Table 2. Statistical assumptions were checked before the test. Normality for each subgroup was assessed visually via histograms, box plots, and Q-Q plots showing only occasional and slight divergence from normality. Levene’s test was performed to evaluate the homogeneity of variances for each subgroup, with the following results: F = 1.92 and *p* = 0.125 for gait cycle time, F = 5.152 and *p* = 0.002 for stance phase, F = 1.164 and *p* = 0.322 for swing phase, F = 5.036 and *p* = 0.002 for relative stance phase, F = 5.044 and *p* = 0.002 for relative swing phase, and F = 5.284 and *p* = 0.001 for cadence. Even though the Levene’s test is significant for four parameters out of six, it is important to indicate that the ANOVA model is just an approximation for the data, and ANOVA assumptions may not be satisfied completely. With normal data but heterogeneous variances, ANOVA is robust for balanced or nearly balanced designs [36,37]. This is due to the fact that Levene’s test relies to a large extent on the sample size. Keppel [38], indeed, suggested that a good rule of thumb is that, if sample sizes are equal, robustness should hold until the largest variance is more than nine times the smallest variance, whose condition is met in this study as variances are comparable among all the subgroups for each parameter.

Some gait parameters (swing phase, relative stance phase, and relative swing phase) do not show a statistically significant interaction between condition and limb, and likewise, do not show the statistical significance of the main effects.

For swing phase it was obtained F (1, 838) = 2.49, *p* = 0.115 for the group-limb interaction, and with the main effects on condition and limb being, respectively, F (1, 838) = 2.325, *p* = 0.128, and F (1, 838) = 0.36, *p* = 0.549. For relative stance phase, we obtained F (1, 838) = 2.588, *p* = 0.108 for the group-limb interaction, and with the main effects on condition and limb being, respectively, F (1, 838) = 0.299, *p* = 0.585, and F (1, 838) = 2.329, *p* = 0.127. For relative swing phase, we obtained F (1, 838) = 2.578, *p* = 0.109 for the group-limb interaction, and with the main effects on condition and limb being, respectively, F (1, 838) = 0.303, *p* = 0.582, and F (1, 838) = 2.333, *p* = 0.127. 

The other parameters (gait cycle time, stance phase, and cadence) showed a significant interaction effect between condition and limb with results being, respectively, F (1, 838) = 5.45, *p* = 0.02, F (1, 838) = 4.005, *p* = 0.046, and F (1, 838) = 7.687, *p* = 0.006. One-way ANOVAs were then performed for those cases considering the simple main effects. Results for the one-way ANOVAs are shown in Table 3. The simple main effects analysis when comparing the players in the post-ACL group with the healthy control group showed that gait parameters obtained from the right (unaffected) leg where not significantly different between the two populations (*p* = 0.46, *p* = 0.355, and *p* = 0.661 for gait cycle time, stance phase, and cadence, respectively). However, when considering the left (affected) leg, statistical significance was observed for gait cycle time and cadence (*p* = 0.011 and *p* = 0.001, respectively). This is also evident from the 95% confidence intervals (CI) for these two cases which, for the left leg gait cycle time were 0.501 to 0.527 s (post-ACL group) and 0.476 to 0.503 s (healthy group), and for the left cadence were 1.943 to 2.055 steps/s (post-ACL group) and 2.079 to 2.2 steps/s (healthy group). However, the effect size, expressed as Cohen’s d (Table 4) calculated for between-limbs and between-group comparisons for each gait-related variable, is observed as very small for all the cases considered, except when analyzing the results of the left leg between healthy and post-ACL subjects (effect size small). Values for Cohen’s d statistics were interpreted as follows: <0.2 for very small, 0.2 to 0.5 for small, 0.5 to 0.8 for medium, 0.8 to 1.3 for large and >1.3 for very large differences [39,40]. A post-hoc analysis showed that, with the effect size obtained (0.15–0.34) and the available sample sizes, the power is too low and the sample size should be increased up to 137 subjects per group to have a power of at least 0.8. 

### 3.2. Machine Learning Model Results

Summary results for all the models are reported in Table 5, while the confusion matrices and the related metrics (sensitivity, specificity, precision, F1-score, and Cohen’s Kappa) are shown in Table 6, Table 7, Table 8, Table 9, Table 10, Table 11 and Table 12, respectively. The overall accuracy is uniform among all the models, between 71.18% and 73.07%.

The kNN model shows an accuracy of 72.34%, with SE of 7.66%, sensitivity was 75.93%, specificity 68.9%, precision 70.63%, F1-score 73.19% and Cohen’s Kappa 0.448. The NB model shows an accuracy of 72.31% (SE: 7.95%), sensitivity 71.95%, specificity 72.8%, precision 72.26%, F1-score 72.1% and Cohen’s Kappa 0.447. The SVM model shows an accuracy of 71.18% (SE: 9.13%), sensitivity 67.9%, specificity 74.5%, precision 72.4%, F1-score 70.08% and Cohen’s Kappa 0.424. The XGB model shows an accuracy of 72.32% (SE: 10.47%), sensitivity 81.8%, specificity 63.07%, precision 68.56%, F1-score 74.6%, and Cohen’s Kappa 0.448. The MLP model shows an accuracy of 73.07% (SE: 8.99%), sensitivity 78.01%, specificity 68.3%, precision 70.79%, F1-score 74.22%, and Cohen’s Kappa 0.462. The stacking model (based on kNN, SVM, NB, XGB, and MLP as base models, and logistic regression as meta-learner) shows an accuracy of 72.84% (SE: 8.95%), sensitivity 77.59%, specificity 68.27%, precision 70.65%, F1-score 73.96%, and Cohen’s Kappa 0.458.

Cohen’s Kappa presents a moderate agreement (between 0.41 and 0.6, according to Landis and Koch [41]) against the expected accuracy for all the considered models. Sensitivity (which identifies the proportion of actual positives correctly identified as such, e.g., the percentage of post-ACL subjects who are correctly identified as part of this class) is higher in XGB, MLP, and the stacking model (up to 81.8%). In contrast, specificity (which measures the proportion of actual negatives correctly identified as such, e.g., the percentage of healthy people who are correctly identified as not having any condition) is between 63.07% and 74.5% with SVM showing the best result. Those results may drive the selection of one model compared to another based on the priorities of athletes and clinicians. Indeed, if there is the requirement to limit the number of false negatives (e.g., the number of subjects in the post-ACL group who are incorrectly classified as healthy) in order to reduce the risk of injury relapse or contralateral ACL injury, then an XGB model should be preferred to the others.

The features selected predominantly across the 10 permutations by the different models are shown in the Excel file in the Supplementary Material. The feature analysis shows that XGB selected overall 15 features, kNN 16, NB 14, SVM 16, and MLP 15. Most of those features are selected among several classifiers. Indeed, 18 out of 250 features have been selected at least once by at least one classifier. In particular, four out of the 18 features were extrapolated from the *y*-axis (anteroposterior axis), six from the *z*-axis (vertical axis), and seven from a combination of the three axes. Moreover, most of the selected features were obtained from derived signals rather than the raw accelerometry and angular rate measurements, in particular, the jerk (12 out of 22 features) and magnitude (five out of 22 features) signals of the 3D acceleration. 

Focusing on the XGB model, which presented the best results, three out of the 15 features were extrapolated from the *y*-axis (anteroposterior axis), five from the *z*-axis (vertical axis), and six from a combination of the three axes. Moreover, most of the selected features were obtained again from jerk (10 out of 15) and magnitude (5 out of 15) signals of the 3D acceleration. 

Finally, all the selected features were obtained from standard time-domain and statistical calculations, such as standard deviation, mean, IQR, RMS, SMA, energy, peak-to-peak, and minimum. 

## 4. Discussion

Return-to-sport following an ACL reconstruction still represents a challenge for clinicians and sport scientists due to the lack of sensitive and objective assessments that could highlight clinically relevant information as to where the athlete is in their journey to recovery. The work presented herein constitutes one of the few studies to investigate the application of wearables sensors in the identification of ACL reconstructed subjects in a group of individuals involved in on-the-field rugby activities so as to classify healthy and post-injury subjects. 

Regarding the first goal of the paper, standard gait parameters from healthy and post-ACL athletes were extrapolated from the collected data and the following statistical analysis demonstrated that some variables (e.g., GCT, and cadence) may be useful to evaluate a long after-effect of the ACL damage, detecting significant differences in ACL-reconstructed vs. healthy players. This confirms the results discussed in [42,43] where a number of male athletes (some of them approx. five years after ACL reconstruction) were asked to run on a treadmill at different speeds to collect kinematic and kinetic variables. Milandri et al. [42] showed that gait velocity may be significantly different between the two cohorts; however, as also indicated in [43], most of the residual long-term differences are evident from ground reaction forces-related metrics and joint moments, which could not be obtained in the studied scenario. In contrast to the works presented in [42,43] which adopted standard optoelectronic or plantar pressure systems, this study only considered the adoption of low-power body-worn motion sensors to guarantee that the assessment could be performed out-of-the lab. Even though recent studies [44,45] have investigated the promising use of IMUs for the estimation of the vertical ground reaction force waveforms via machine learning approaches, their application to on-the-field conditions still needs to be confirmed, and thus, those metrics have not been considered in this study. 

A power analysis showed that, based on standard alpha and beta levels of 0.05 and 0.8, respectively, a large effect size (standardized by Cohen as 0.8 [39,40]) would require a minimum sample size of 26 subjects per group. The effect size in the experiment was observed as very small for all the cases considered, except when analyzing the results of the injured leg between healthy and post-ACL subjects (effect size small). A post-hoc analysis showed that, with the effect size obtained (0.15–0.34) and the available sample sizes, the power is too low, and hence the sample size should be increased up to 137 subjects per group to have a power of at least 0.8. However, no study available in literature on the investigated topic fulfills this criteria, and even the ones meeting the criteria of the 26 subjects per group are scarce. 

Therefore, even though some statistical significance was detected in the analysis, the small observed power and effect size do not provide enough confidence that the difference seen between groups for those variables was a real observed effect and, as a result, further larger studies should be performed.

Regarding the second goal of the paper, the importance of addressing gait pattern classification in biomechanics, and, in particular, in defining which parameters can distinguish between post-ACL subjects from healthy controls is well-known in literature [46,47,48]. While those works relied on gait metrics, recent works published by Wu et al. [49] and Richter et al. [50] also considered the application of machine learning models for the discrimination between ACL deficient and healthy subjects. Nevertheless, all those studies were carried out in gait laboratories using gold-standard marker-based optoelectronic systems, thus limiting the applicability of those insights to real-world use cases. 

Moreover, standard gait parameters may not be relevant or applicable during typical sport movements, such as cutting manoeuvres or jumping, causing an unreliable step-detection. Therefore, a data-driven approach based on machine learning models and motion data has also been developed with the goal to objectively discriminate between the two cohorts. This method, as it is independent of the step detection, may be more robust and accurate than standard gait analysis for on-the-field scenarios, which is a concept already defined for falling risk classification [51]. 

The final outcome of a MLP classifier showed an overall accuracy on the test dataset of 73.07%, which is only slightly better compared to the other models investigated (worst accuracy: 71.18% for SVM). The standard error for all the models was large (>7%) which is due to the limited number of subjects involved in the test dataset. 

Even though accuracy may seem similar for all the approaches, those models show different performances when looking at the misclassifications and related metrics (e.g., sensitivity, specificity). Limiting the number of false negatives rather than the false positives may be more appealing for athletes and coaches in order to minimize possible re-injuries, and thus models with large sensitivity (81.8% for XGB) may be more helpful for the end-users when out-of-the-lab. 

Observing the features repeatedly selected by the XGB model and the other classifiers, it is evident that >50% of the chosen features are related to the sagittal plane. This insight confirms the results reported in [7,52] which indicated that most of the alterations of interest take place in the sagittal plane. Moreover, >50% of those features were connected with the jerk of the 3D acceleration again confirming the results observed in [53]. The jerk, indeed, has been found to characterize dynamic movement at the knee joint and it has been successfully used as an indicator of the lack of stable neuromuscular control or structural instability, often observed in ACL subjects, because this movement correlated with patient reports of instability [53]. Finally, all the selected features were obtained from standard time-domain and statistical features, such as standard deviation, mean, IQR, RMS, SMA, energy, peak-to-peak, and minimum. 

The considered features were different from those used in the state-of-the-art for similar problems; this was due to the underlying concept of building fully data-driven models which do not rely on traditional biomechanical parameters and step detection, hence trading model performance vs. its interpretability. For example, Patterson et al. [21] considered gyroscope-extracted features (such as shank rotation rate and its variance, and shank rate of change at different moments in the gait cycle) besides the conventional gait temporal variables. Setuain et al. [22] instead adopted jumping-related biomechanical features (e.g., vertical velocity, mechanical efficiency ratio). However, in both cases [21,22], no machine learning model was developed to discriminate between the two populations of interest. Wu et al. [49] built a neural network based on the features extrapolated from the 3D phase space reconstruction of the knee mechanics during the internal-external rotation, and flexion-extension, antero-poster, and proximal-distal translations while walking on a treadmill. On the other hand, Richter et al. [50] considered a wide range of biomechanical features, including ground reaction forces and impulses, center of mass velocity and power in pelvis, hip, knee, and ankle, as well as joint angles of the ankle, knee, hip, pelvis, thorax, and thorax on pelvis in sagittal, frontal, and transversal planes, joint angular velocities of the ankle, knee, hip, pelvis, thorax, and thorax on pelvis in sagittal, frontal, and transversal plane, joint powers, moments, work, and impulse of ankle, knee, hip, and pelvis in sagittal, frontal, and transversal plane, time, and the rotation foot angle to pelvis. Moreover, different exercises were also tested, such as double leg countermovement jump, single leg countermovement jump, double leg drop jump, single leg drop jump, hurdle hop, single leg hop, as well as planned and unplanned change of direction. The analysis showed that during an unplanned change of direction task (as the one implemented in the present investigation) the highest achieved accuracy was 67% with the best model based on discriminant analysis relying on vertical centre of mass velocity and hip flexion moment, hence in line with the performance results reported in this paper. It was also observed that double leg countermovement jump and double leg drop jump were the exercises that show the highest accuracy in the discrimination between post-ACL and healthy athletes with 82% and 87% accuracy, respectively. However, Richter et al. [50] recruited the post-ACL population only nine months after ACL reconstruction, and both [49,50] relied on optoelectronic systems and force platforms. Finally, Mandalapu et al. [24] adopted inertial sensors on the ankles, wrists, and sacrum on athletes while walking and jogging on a treadmill. The features considered were fully data-driven, e.g., phase slope index and pairwise causality matrix, and managed to achieve good performance in the discrimination task using auto multi-layer perceptron and neural network with the highest area under the curve of 0.76 and Cohen’s Kappa of 0.53 (again, in line with the results presented in this paper). Interestingly, Mandalapu et al. [24] also reported slightly better model performance in female athletes compared to male athletes.

The present study is one of the few in literature which adopted motion sensors for studying the discrimination between post-ACL and healthy athletes and, to the best of the authors’ knowledge, the first to use a combination of wearable sensors and machine learning models in field-settings. The results of this study clearly show that motion sensors can distinguish between players with ACL-reconstructed knee and healthy players even after 5–10 years following the injury, despite the previously injured athletes being deemed fully recovered. This is a promising result that could suggest the reliable use of these sensors in real training environments, thus supporting the decision-making process of physiotherapists, medical staff, and sport scientists in their practice. 

This study was conducted in a free-living environment using only motion sensors, therefore no gold-standard optical motion tracking system was adopted for monitoring the athletes’ biomechanical conditions. This gold-standard assessment would have provided further clarification regarding the observed significant differences in the stance phase and cadence parameters estimated from both limbs in the healthy group. However, it is not unusual to observe gait asymmetries also in healthy subjects when running, as already indicated in literature in [54,55], with the main reasons being indicated as the running speed, the runner’s running experience, and fatigue. 

Given that the analyzed rugby players were not part of a team and the previously injured subjects were monitored by different independent physiotherapists, the consideration of them being deemed fully recovered was based exclusively on the reports from the athletes’ clinicians. 

The analysis carried out in this study did not include subject-related features (e.g., height, weight) in order to develop subject-independent models based exclusively on data collected from the wearable sensors and the movement mechanics. Further analysis should be performed to highlight the impact that subject-related features may have on the model performance. 

Moreover, gender and small sample size are other limitations of the study which may limit the generalizability of the results. Given the novelty of the study, the present investigation was designed as a pilot proof-of-concept; larger cohort will need to be recruited in the future to confirm those results as shown by the power analysis. Furthermore, the collection of a larger dataset could enable the possibility to adopt more powerful techniques associated to deep learning. The impact of different feature selection approaches on the model performance could also be investigated [56]. Also, it is unknown if the post-ACL subjects reported similar asymmetries before the injury or any other time beside the test session in which they were recorded. Finally, despite the numerous tests available in return-to-sport protocols, this study focused specifically on cutting maneuvers, because of their high-risk mechanics and relations to ACL injuries [6,57], even though additional tasks should also be considered in future studies [50].

Therefore, further larger scale and longitudinal studies should be defined to confirm these insights, in a less controlled environment and adopting additional sensing technology (e.g., pressure insoles, surface electromyography, full-body motion sensors) for model validation. 

## 5. Conclusions

This paper investigated the possibility of applying motion sensors on the lower limbs for the identification of post-ACL subjects (approx. 5–10 years following injury) in a group of athletes involved in on-the-field rugby activities. Twelve participants were recruited (six healthy and six post-ACL) and asked to perform running and change-of-direction activities while wearing devices on their thighs and shanks. Time- and frequency-domain features were extracted from the raw signals. 

Standard gait analysis parameters shows that GCT and cadence may be useful for discriminating the two cohorts. An automated data-driven method based on machine learning models shows that it is possible to correctly classify subjects with an accuracy of 73.07% (MLP) and sensitivity of 81.8% (XGB). The results of this study suggest the feasibility to use body-worn motion sensors and machine learning approaches for the identification of post-ACL gait patterns in athletes performing sport tasks on-the-field even a number of years after the injury occurred. The adoption of this method may provide clinicians and sport scientists relevant information regarding the RTS assessment of injured athletes and the related rehabilitation programme. 

## Figures and Tables

**Table 1:** Participants Characteristics.

Subjects #	Injured Leg	Time since Injury (years)	Height (cm)	Weight (Kg)	Treatment
1	Left	5	179	90	ACLR
2	Left	6	190	106	ACLR (twice)
3	Left	10	180	108	ACLR
4	Left	5	187	75	ACLR
5	Left	5	173	78	ACLR
6	Left	6	185	78	ACLR
**Mean (SD)**		6.2 (1.9)	182.3 (6.2)	89.2 (14.7)	
7	-	-	178	70	-
8	-	-	185	104	-
9	-	-	189	94	-
10	-	-	175	85	-
11	-	-	180	97	-
12	-	-	190	95	-
**Mean (SD)**			182.8 (6.1)	90.8 (11.9)	
**Overall Mean (SD)**			182.6 (5.9)	90 (12.8)	

**Table 2:** Gait Temporal Parameters—Descriptive Statistics.

Group	Limb	GCT [s]—Mean (SD)	STP [s]—Mean (SD)	SWP [s]—Mean (SD)	rSTP [%]—Mean (SD)	rSWP [%]—Mean (SD)	Cadence [steps/s]—Mean (SD)
Post-ACL	Left	0.514 (0.09)	0.232 (0.065)	0.281 (0.046)	44.75 (7.52)	55.25 (7.52)	1.99 (0.36)
	Right	0.503 (0.088)	0.229 (0.07)	0.274 (0.045)	44.7 (8.84)	55.3 (8.84)	2.05 (0.39)
	Total	0.5083 (0.089)	0.23 (0.067)	0.277 (0.045)	44.72 (8.2)	55.27 (8.2)	2.02 (0.37)
Healthy	Left	0.49 (0.1)	0.219 (0.08)	0.271 (0.047)	43.4 (9.81)	56.58 (9.81)	2.14 (0.49)
	Right	0.509 (0.101)	0.235 (0.07)	0.274 (0.054)	45.35 (9.65)	54.64 (9.65)	2.03 (0.39)
	Total	0.499 (0.102)	0.227 (0.08)	0.272 (0.051)	44.38 (9.76)	55.61 (9.76)	2.08 (0.45)
Overall	Left	0.502 (0.096)	0.226 (0.07)	0.276 (0.047)	44.13 (8.67)	55.87 (8.67)	2.06 (0.43)
	Right	0.506 (0.095)	0.232 (0.073)	0.274 (0.049)	45 (9.22)	54.99 (9.22)	2.04 (0.39)
	Total	0.504 (0.095)	0.229 (0.073)	0.275 (0.048)	44.57 (8.96)	55.43 (8.96)	2.05 (0.41)

**Table 3:** One-way ANOVAS—Summary Results (F and *p*-values).

	GCT	STP	Cadence
Post-ACL vs. Healthy (Left Leg)	F = 6.478, *p* = **0.011**	F = 3.633, *p* = 0.057	F = 11.267, *p* = **0.001**
Post-ACL vs. Healthy (Right Leg)	F = 0.547, *p* = 0.46	F = 0.859, *p* = 0.355	F = 0.192, *p* = 0.661
Left vs. Right Leg (Post-ACL Group)	F = 1.724, *p* = 0.19	F = 0.344, *p* = 0.558	F = 2.457, *p* = 0.118
Left vs. Right Leg (Healthy Group)	F = 3.649, *p* = 0.057	F = 4.239, *p* = **0.04**	F = 5.058, *p* = **0.025**

In bold: statistically significant difference (*p* < 0.05).

**Table 4:** Effect sizes calculations.

	GCT	STP	SWP	rSTP	rSWP	Cadence
Post-ACL vs. Healthy (Left Leg)	0.252	0.178	0.215	0.15	0.15	0.34
Post-ACL vs. Healthy (Right Leg)	0.06	0.08	0	0.07	0.07	0.05
Post-ACL vs. Healthy (Both Legs)	0.09	0.04	0.1	0.03	0.03	0.14
Left vs. Right Leg (Post-ACL Group)	0.12	0.04	0.15	0.006	0.006	0.16

**Table 5:** Models Performance (Accuracy).

	kNN	NB	SVM	XGB	MLP	Stacking
**Training Accuracy (SE)**	81.53% (4.81%)	75.90% (6.38%)	76.32% (7.07%)	77.45% (6.52%)	76.78% (5.98%)	77.27% (5.98%)
**Test Accuracy (SE)**	72.34% (7.66%)	72.31% (7.95%)	71.18% (9.13%)	72.32% (10.47%)	73.07% (8.99%)	72.84% (8.95%)

**Table 6:** Confusion matrix for kNN. (TP: true positive, TN: true negative, FN: false negative, FP: false positive).

		Predicted Condition	
		Predicted Condition Positive	Predicted Condition Negative
**True Condition**	**Condition Positive**	TP = 2345	FN = 743
	**Condition Negative**	FP = 975	TN = 2161

**Table 7:** Confusion matrix for NB. (TP: true positive, TN: true negative, FN: false negative, FP: false positive).

		Predicted Condition	
		Predicted Condition Positive	Predicted Condition Negative
**True Condition**	**Condition Positive**	TP = 2222	FN = 866
	**Condition Negative**	FP = 853	TN = 2283

**Table 8:** Confusion matrix for SVM. (TP: true positive, TN: true negative, FN: false negative, FP: false positive).

		Predicted Condition	
		Predicted Condition Positive	Predicted Condition Negative
**True Condition**	**Condition Positive**	TP = 2097	FN = 991
	**Condition Negative**	FP = 799	TN = 2337

**Table 9:** Confusion matrix for XGB. (TP: true positive, TN: true negative, FN: false negative, FP: false positive).

		Predicted Condition	
		Predicted Condition Positive	Predicted Condition Negative
**True Condition**	**Condition Positive**	TP = 2526	FN = 562
	**Condition Negative**	FP = 1158	TN = 1978

**Table 10:** Confusion matrix for MLP. (TP: true positive, TN: true negative, FN: false negative, FP: false positive).

		Predicted Condition	
		Predicted Condition Positive	Predicted Condition Negative
**True Condition**	**Condition Positive**	TP = 2409	FN = 679
	**Condition Negative**	FP = 994	TN = 2142

**Table 11:** Confusion matrix for stacking. (TP: true positive, TN: true negative, FN: false negative, FP: false positive).

		Predicted condition	
		Predicted Condition Positive	Predicted Condition Negative
**True Condition**	**Condition Positive**	TP = 2396	FN = 692
	**Condition Negative**	FP = 995	TN = 2141

**Table 12:** Models Performance (Sensitivity, Specificity, Precision, F1-score, Cohen’s Kappa).

	kNN	NB	SVM	XGB	MLP	Stacking
**Sensitivity**	75.93	71.95	67.9	81.8	78.01	77.59
**Specificity**	68.9	72.8	74.5	63.07	68.3	68.27
**Precision**	70.63	72.26	72.4	68.56	70.79	70.65
**F1-Score**	73.19	72.1	70.08	74.6	74.22	73.96
**Cohen’s Kappa**	0.448	0.447	0.424	0.448	0.462	0.458

## Supplementary Materials

The following are available online at https://www.mdpi.com/1424-8220/20/11/3029/s1, Table S1: Features Extracted; Table S2: Hyper-parameters Grid search.
