# Supplementary material for: Motion Sensors-Based Machine Learning Approach for the Identification of Anterior Cruciate Ligament Gait Patterns in On-the-Field Activities in Rugby Players
Source: Sensors (Basel). 2020 May 27;20(11):3029. doi: 10.3390/s20113029 (PMC7309071; doi:10.3390/s20113029)
Supplement: Supplementary file 1 [file sensors-20-03029-s001.zip › Supplementary Material/Supplementary_Material_features.docx.pdf]

Supplementary Material. Features Extracted

Table S1. Features Extracted.

Feature type	Signals to which it is applied	Number of features
Mean	Gyroscope (x, y, z), Body-frame acceleration (x, y, z), Gravity-frame acceleration (x, y, z), Jerk (x, y, z), Magnitude of acceleration	13
Standard deviation	Gyroscope (x, y, z), Body-frame acceleration (x, y, z), Gravity-frame acceleration (x, y, z), Jerk (x, y, z), Magnitude of acceleration	13
Skewness	Gyroscope (x, y, z), Body-frame acceleration (x, y, z), Gravity-frame acceleration (x, y, z), Jerk (x, y, z), Magnitude of acceleration	13
Kurtosis	Gyroscope (x, y, z), Body-frame acceleration (x, y, z), Gravity-frame acceleration (x, y, z), Jerk (x, y, z), Magnitude of acceleration	13
Root Mean Square (RMS)	Gyroscope (x, y, z), Body-frame acceleration (x, y, z), Gravity-frame acceleration (x, y, z), Jerk (x, y, z), Magnitude of acceleration	13
Minimum	Gyroscope (x, y, z), Body-frame acceleration (x, y, z), Gravity-frame acceleration (x, y, z), Jerk (x, y, z), Magnitude of acceleration	13
Maximum	Gyroscope (x, y, z), Body-frame acceleration (x, y, z), Gravity-frame acceleration (x, y, z), Jerk (x, y, z), Magnitude of acceleration	13
Coefficient of Variation (CV)	Gyroscope (x, y, z), Body-frame acceleration (x, y, z), Gravity-frame acceleration (x, y, z), Jerk (x, y, z), Magnitude of acceleration	13
Peak-to-peak	Gyroscope (x, y, z), Body-frame acceleration (x, y, z), Gravity-frame acceleration (x, y, z), Jerk (x, y, z), Magnitude of acceleration	13
Interquartile Range (IQR)	Gyroscope (x, y, z), Body-frame acceleration (x, y, z), Gravity-frame acceleration (x, y, z), Jerk (x, y, z), Magnitude of acceleration	13
Pairwise correlation	Gyroscope (x, y, z), Body-frame acceleration (x, y, z), Gravity-frame acceleration (x, y, z), Jerk (x, y, z)	12
Signal Magnitude Area (SMA)	Gyroscope (x, y, z), Body-frame acceleration (x, y, z), Gravity-frame acceleration (x, y, z), Jerk (x, y, z)	4

Energy	Gyroscope (x, y, z), Body-frame acceleration (x, y, z), Gravity-frame acceleration (x, y, z), Jerk (x, y, z), Magnitude of acceleration	13
Jerk-based smoothness measure [23]	Jerk (x, y, z)	3
Spectral arc length (SPARC) [25]	Gyroscope (x, y, z), Acceleration (x, y, z)	2
Sample entropy [24] (6 features per each signal)	Gyroscope (x, y, z), Acceleration (x, y, z)	36
Dominant frequency	Gyroscope (x, y, z), Acceleration (x, y, z), Jerk (x, y, z), Magnitude of acceleration	10
Spectral centroid	Gyroscope (x, y, z), Acceleration (x, y, z), Jerk (x, y, z), Magnitude of acceleration	10
Spectral edge frequency	Gyroscope (x, y, z), Acceleration (x, y, z), Jerk (x, y, z), Magnitude of acceleration	10
Harmonic ratio	Gyroscope (x, y, z), Acceleration (x, y, z), Jerk (x, y, z), Magnitude of acceleration	10
Index of harmonicity	Gyroscope (x, y, z), Acceleration (x, y, z), Jerk (x, y, z), Magnitude of acceleration	10
**Total Number of Features**		**250**

Supplementary Material. Hyper-parameters grid search

Table S2. Hyper-parameters Grid search.

**Model**	**Hyper-parameters**
Naïve Bayes	K = (1, 10) for Select K Best
kNN	Weights = ['uniform' , 'distance']<br>Algorithm = ['auto' , 'ball_tree' , 'kd_tree' , 'brute']<br>N_neighbors = (1, 10)<br>K = (1, 10) for Select K Best
SVM	Kernel = ['linear' , 'poly' , 'rbf' , 'sigmoid']<br>C = Real(1e-6, 1, 'log-uniform')<br>gamma = ['scale' , 'auto']

	K = (1, 10) for Select K Best
XGB	Learning_rate = Real(0.0001, 0.3, ‘uniform’)<br>N_estimators = (10, 10000)<br>Max_depth = (3, 10)<br>Max_child_weight = (1, 6)<br>Gamma = Real(20, 50, ‘uniform’)<br>Subsample = Real(0.5, 0.9, ‘uniform’)<br>Colsample_bytree = Real (0.5, 0.9, ‘uniform’)<br>Reg_alpha = Real(1e-5, 100, ‘log-uniform’)<br>K = (1, 10) for Select K Best
MLP	Hidden_layer_sizes = (1, 100)<br>Activation = [‘tanh’, ‘relu’]<br>Solver = [‘sgd’, ‘adam’]<br>Alpha = Real(0.1, 5, ‘log-uniform’)<br>Learning_rate = [‘constant’ , ‘adaptive’]<br>Early_stopping = [‘True’ , ‘False’]<br>K = (1, 10) for Select K Best
Stacking	Every combination of three classifiers (NB, kNN, SVM, XGB, MLP) as base-classifiers<br>C = Real(0.001, 1000, ‘log-uniform’) for logistic regression as meta-classifier<br>K = (1, 10) for Select K Best
